# LTX-315 sequentially promotes lymphocyte-independent and lymphocyte-dependent antitumor effects

**DOI:** 10.15698/cst2019.11.204

**Published:** 2019-10-14

**Authors:** Hsin-Wei Liao, Christopher Garris, Christina Pfirschke, Steffen Rickelt, Sean Arlauckas, Marie Siwicki, Rainer H. Kohler, Ralph Weissleder, Vibeke Sundvold-Gjerstad, Baldur Sveinbjørnsson, Øystein Rekdal, Mikael J. Pittet

**Affiliations:** 1 Center for Systems Biology, Massachusetts General Hospital Research Institute, Harvard Medical School, Boston, MA, USA.; 2 David H. Koch Institute for Integrative Cancer Research, Massachusetts Institute of Technology, Cambridge, MA, USA.; 3 Lytix Biopharma, Oslo, Norway.; 4 Department of Medical Biology, University of Tromsø, Tromsø, Norway.; # These authors contributed equally.

**Keywords:** cancer, immunotherapy, oncolytic peptide, CD8+ T cells, melanoma, sarcoma

## Abstract

LTX-315 is an oncolytic peptide that has antitumor efficacy in mice grafted with various tumor cell lines and is currently being tested in phase II clinical trials. Here we aimed to further evaluate LTX-315 in conditional genetic mouse models of cancer that typically resist current treatment options and to better understand the drug's mode of action *in vivo*. We report LTX-315 mediates profound antitumor effects against *Braf-* and *Pten*-driven melanoma and delays the progression of *Kras-* and *P53-*driven soft tissue sarcoma in mice. Additionally, we show in melanoma that LTX-315 triggers two sequential phases of antitumor response. The first phase of response, which begins within minutes of drug delivery into tumors, is defined by disrupted tumor vasculature and decreased tumor burden and occurs independently of lymphocytes. The second phase of response, which continues over weeks, is defined by long-term alteration of the tumor microenvironment; the changes induced by LTX-315 are most notably characterized by CD8+ T cell infiltration. We further show that these CD8+ T cells are involved in suppressing melanoma outgrowth in mice and report similar CD8+ T cell infiltration following LTX-315 treatment in melanoma and sarcoma patients. Taken together, these findings reveal LTX-315's multiple antitumor effects, including disrupting the tumor vasculature and promoting the conversion of poorly immunogenic tumors into ones that display antitumor T cell immunity.

## INTRODUCTION

LTX-315 is a chemically modified 9-mer cationic peptide that derives from bovine lactoferrin [1]. This peptide is referred to as oncolytic, due to its potent cytotoxic activity against a variety of murine and human cancer cell lines. For example, LTX-315 can kill cancer cells by disrupting cell membranes and targeting mitochondria [2]. This mechanism of action is relevant to cancer immunotherapy because it promotes the release of damage-associated molecular patterns [3], which are linked with immunogenic cell death [4]. Consequently, LTX-315 is considered therapeutically promising for its ability to not only kill tumor cells but also do so in a way that can promote antitumor immunity. LTX-315 has relatively high plasma protein binding capacity and a human plasma half-life of 15 min. Based on these properties, LTX-315 is being developed as a new anticancer agent for intratumoral administration [1] and is currently being evaluated clinically as a first-in-class oncolytic peptide-based local immunotherapy [5].

Studies in mouse models have shown that intratumorally administering LTX-315 in intradermally established B16F10 melanomas results in complete tumor regression in the majority of animals treated [6]. Furthermore, cured animals are protected from B16F10 rechallenge, indicating that LTX-315 treatment can induce adaptive antitumor immune responses [6]. LTX-315 administration also regresses established murine MCA205 sarcomas. In these mice, the treatment reprograms the MCA205 tumor microenvironment in part by increasing the local abundance of cytotoxic CD8+ T cells and by reducing the frequency of immunosuppressive regulatory T cells [7]. Similarly, in rat models, LTX-315 treatment can successfully eliminate intradermally established rTMSC fibrosarcomas and trigger protective and systemic antitumor immunity [8].

Considering that these previous experimental studies identified LTX-315's ability to control cancer in mice grafted with various tumor cell lines, we sought to establish whether the drug is equally effective in conditional genetic mouse models of cancer and determine its specific mechanisms of action. We used *Braf*^*V600E*^*/Pten*^*−/−*^ (BP) mice, which develop spatially restricted melanoma upon tamoxifen application [9], and *Kras*^*LSL-G12D/+*^*;p53*^*fl/fl*^ (KP) mice, which develop spatially restricted soft tissue sarcoma upon intramuscular administration of adenovirus expressing Cre (AdCre) [10, 11]. In both models, cancer cells derive from somatic cells that are transformed in their normal tissue microenvironment and progress to high-grade tumors that are poorly infiltrated by T cells and typically resist prescribed chemo- and immunotherapeutic treatments. This is important, considering that many human tumors, including melanomas and sarcomas, do not benefit from current treatments such as immunotherapies in part because T cells are excluded from the vicinity of cancer cells [12–14].

Here, we report that LTX-315 delays tumor progression substantially in these genetic mouse models. Using melanoma models, we also identify two sequential phases of antitumor response triggered by LTX-315: the first phase is lymphocyte independent and defined by rapid disruption of the tumor vasculature, the second phase is defined by long-term alteration of the tumor microenvironment and infiltration by CD8+ T cells, which display antitumor functions. Transition from a ‘cold' to a ‘hot' tumor microenvironment, infiltrated by cytotoxic T cells, was also observed in melanoma and sarcoma patients treated with LTX-315.

## RESULTS

### LTX-315's antitumor effects in melanoma and soft tissue sarcoma mouse models

**Figure 1:**
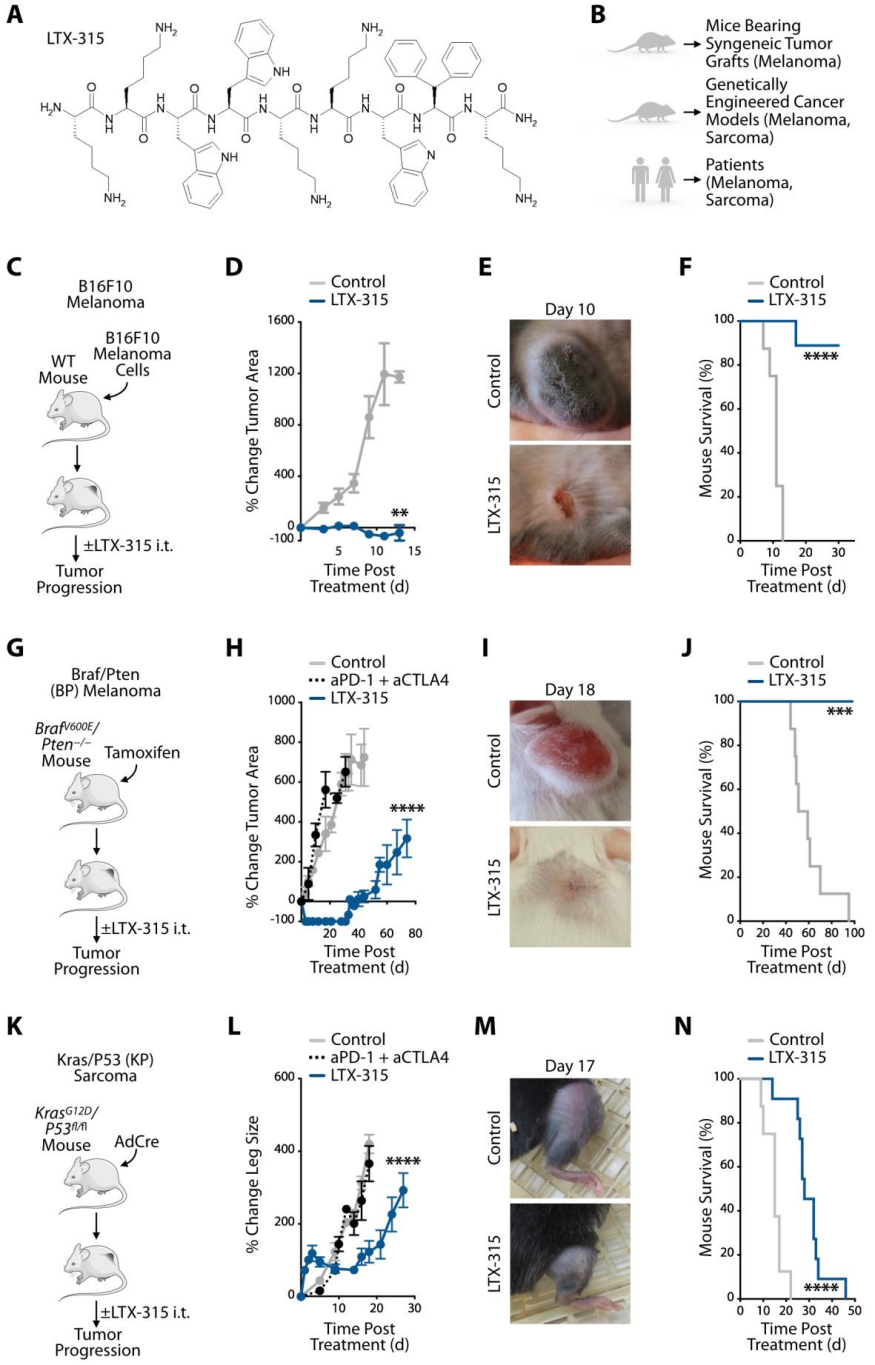
FIGURE 1: LTX-315 controls tumor growth and improves survival in conditional genetic melanoma and soft tissue sarcoma mouse models. **(A)** Chemical structure of oncolytic peptide LTX-315 (KKWWKKWDipK where Dip is β-diphenylalanine, which promotes stiffness and a rigid peptide structure). **(B)** Experimental approaches used to study LTX-315 effects in melanoma and sarcoma mouse models and in cancer patients. **(C)** Schematic of B16F10 melanoma experiments: C57BL/6 mice bearing B16F10 melanoma tumor grafts were either treated intratumorally (i.t.) with LTX-315 or left untreated. **(D)** Change in B16F10 tumor area of LTX-315-treated or control mice relative to pre-treatment baseline. n = 8 to 9 mice/group. **(E)** Representative images of B16F10 tumors either on day 10 after LTX-315 treatment or from untreated mice. **(F)** Kaplan-Meier survival analysis of B16F10 tumor-bearing mice treated with LTX-315 (blue) or left untreated (gray). n = 8 to 9 mice/group. **(G)** Schematic of BP melanoma experiments: *Braf*^*V600E*^*/Pten*^*−/−*^ (BP) mice subjected to tamoxifen to produce tumors were either treated intratumorally (i.t.) with LTX-315 or left untreated. **(H)** Change in BP tumor area of LTX-315-treated or control mice relative to pre-treatment baseline. Additional mice received immune checkpoint blockade treatment with anti-PD1 and anti-CTLA-4 antibodies (aPD-1 + aCTLA4, black). n = 4 to 7 mice/group. **(I)** Representative images of BP tumors either on day 18 after LTX-315 treatment or from untreated mice. **(J)** Kaplan-Meier survival analysis of BP tumor-bearing mice treated with LTX-315 (blue) or left untreated (gray). n = 5 to 8 mice/group. **(K)** Schematic of KP soft tissue sarcoma experiments: *Kras*^*G12D*^*/p53*^*fl/fl*^ (BP) mice subjected to intramuscular leg injection with Adenovirus expressing Cre recombinase (AdCre) to produce tumors were either treated intratumorally (i.t.) with LTX-315 or left untreated. **(L)** Change in leg size of LTX-315-treated or untreated KP mice relative to pre-treatment leg size. Additional mice received immune checkpoint blockade treatment (aPD-1 + aCTLA4, black) intraperitoneally. n = 5 to 8 mice/group. **(M)** Representative images of KP tumors either on day 17 after LTX-315 treatment or from untreated mice. **(N)** Kaplan-Meier survival analysis of KP tumor-bearing mice treated with LTX-315 (blue) or left untreated (gray). n = 8 to 11 mice/group. Results are expressed as mean ± SEM. **p < 0.01; ***p < 0.001; ****p < 0.0001. Abbreviations are as follows: d = day.

We sought to study responses to LTX-315 **(Fig. 1A)** in various experimental models, including mice bearing syngeneic tumors and genetically engineered mouse models, as well as in cancer patients **(Fig. 1B)**. Initially, we evaluated wild-type mice grafted with syngeneic B16F10 melanoma cells **(Fig. 1C** and **Fig. S1)**. Intratumoral LTX-315 treatment dramatically decreased B16F10 tumor burden **(Fig. 1D-E)** and improved overall mouse survival **(Fig. 1F)** when compared to untreated mice. These findings accord with previous studies showing that LTX-315 can effectively prevent cancer progression in mice grafted with various tumor cell lines [15].

We also evaluated LTX-315 treatment in the genetically induced *Braf*^*V600E*^*/Pten*^*−/−*^ (BP) melanoma mouse model **(Fig. 1G** and **Fig. S1)**. LTX-315 injection within established BP tumors led to macroscopic tumor mass disappearance within days **(Fig. 1H, I)**. LTX-315-mediated tumor control was not only profound but also lasted for more than four weeks, at which time tumors eventually started to grow again **(Fig. 1H)**. By contrast, systemic treatment with anti-PD-1 and anti-CTLA-4 mAbs failed to control BP tumor progression **(Fig. 1H)**. A Kaplan-Meier study of BP mice treated or not with LTX-315, and followed for ~100 days after treatment, showed improved overall survival of the LTX-315-treated mice **(Fig. 1J)**.

We then extended our investigation of LTX-315 therapy to the genetically induced *Kras*^*LSL-G12D/+*^*;p53*^*fl/fl*^ (KP) soft tissue sarcoma model **(Fig. 1K** and **Fig. S1)**. Intratumoral LTX-315 treatment significantly delayed KP tumor progression, which systemic treatment with anti-PD-1 and anti-CTLA-4 mAbs failed to do **(Fig. 1L, M)**. LTX-315-mediated tumor control typically lasted up to day ~15 **(Fig. 1L)**. By contrast, tumors in mice that were either left untreated or that received anti-PD-1 and anti-CTLA-4 continuously grew. Additionally, a Kaplan-Meier study of KP mice demonstrated increased overall survival of the LTX-315-treated cohort **(Fig. 1N)**.

The observation that LTX-315 significantly delays KP tumor progression parallels our findings from the BP model and indicates LTX-315's ability to mediate antitumor effects against different genetically induced cancer models and tumors with distinct tissues of origin. We also noted differences when comparing the KP and BP models, suggesting specific drug activities in these models. First, tumor control following LTX-315 treatment was less durable in the KP sarcoma model. Second, we observed a transient increase in measurable KP (but not BP) tumor burden at the LTX-315 injection site. The change was already noticeable one day after treatment and subsided for around three to four days, at which time tumor burden visibly decreased. The reasons for these differences remain uncharacterized.

### LTX-315's first phase of response

**Figure 2:**
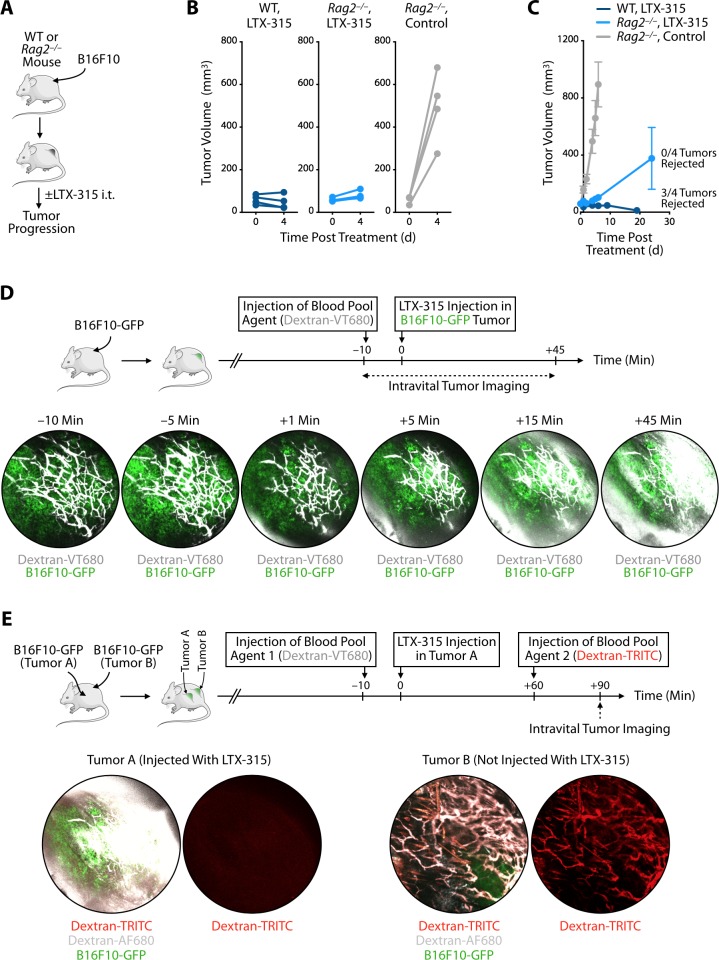
FIGURE 2: LTX-315's initial phase of antitumor response is defined by rapid disruption of the tumor vasculature and occurs independently of lymphocytes. **(A)** Schematic of B16F10 melanoma experiments: *Rag2*^*–/–*^ or control wild type (WT) mice bearing B16F10 melanoma tumor grafts were either treated intratumorally (i.t.) with LTX-315 or left untreated. **(B)** B16F10 tumor volumes immediately before (d0) and four days after LTX-315 treatment (d4) in individual WT mice (dark blue) and *Rag2*^*–/–*^ mice (light blue). Untreated *Rag2*^*–/–*^ mice (grey) were used as controls; n = 4 mice/group. **(C)** Cumulative B16F10 tumor volumes over time of mice treated as in (B). **(D)** Intravital microscopy of a vasculature agent (Dextran-VT680, white) at different time points before and after intratumoral LTX-315 injection in mice bearing B16F10-GFP melanoma tumors (green). **(E)** Intravital microscopy of two vasculature agents (Dextran-VT680, white; Dextran-TRITC, red) in mice bearing two B16F10-GFP melanoma tumors (Tumors A and B) and after intratumoral LTX-315 injection in Tumor A only. Dextran-VT680 was injected 10 min before LTX-315 administration, whereas Dextran-TRITC was given 60 min later. Abbreviations are as follows: d = day.

Considering LTX-315's ability to control tumors rapidly (i.e. within the first few days) in the models tested, we sought to further investigate the process leading to initial tumor elimination using the melanoma mouse models. LTX-315 treatment has been shown to induce adaptive antitumor immune responses [8]; yet, the kinetics of tumor elimination we observed suggested that the initial antitumor events triggered by LTX-315 occur too early to depend on a response mediated by adaptive immune lymphocytes. To test this hypothesis, we compared the effects of LTX-315 treatment in either wild-type or *Rag2*^*–/–*^ (lymphocyte deficient) mice bearing B16F10 melanoma tumors **(Fig. 2A** and **Fig. S1)**. We found that LTX-315 controlled tumor burden equally during the first four days in both mouse cohorts, whereas a control group confirmed tumor growth in un-treated *Rag2*^*–/–*^ mice **(Fig. 2B)**. At later time points, however, wild-type mice treated with LTX-315 typically rejected their tumors, whereas *Rag2*^*–/–*^ mice did not **(Fig. 2C)**. These findings not only confirm that LTX-315 can trigger long-term adaptive immune responses but also indicate the drug's capacity to mediate rapid antitumor effects that are independent of lymphocytes.

With the goal to further explore the events that follow intratumoral LTX-315 administration in melanoma, we used intravital imaging to study the microenvironment of B16F10 tumors growing in a skinfold window chamber. Specifically, mice were implanted with B16F10 tumors expressing green fluorescent protein (GFP) and injected intravenously with the blood pool agent Dextran-VT680 around ten minutes before LTX-315 administration in B16F10-GFP tumors **(Fig. 2D)**. Longitudinal imaging revealed rapid changes of Dextran-VT680 biodistribution following LTX-315 treatment. Most notably, imaging the tumor stroma showed leakage of the blood pool agent as early as 15 minutes after LTX-315 treatment, suggesting the oncolytic peptide had instigated a profound and rapid disruption of the tumor vasculature **(Fig. 2D)**.

To further investigate the effects of LTX-315 treatment on the local tumor microenvironment, we used mice bearing B16F10-GFP tumors at two distinct locations, termed Tumor A and Tumor B. Only Tumor A was treated with LTX-315. Also, these mice received two blood pool agents at distinct times: Dextran-VT680 (Blood Pool Agent 1) was given ten minutes before LTX-315 treatment, whereas Dextran-TRITC (Blood Pool Agent 2) was given 60 minutes after LTX-315 treatment **(Fig. 2E)**. Intravital imaging performed 90 minutes after LTX-315 injection showed leakage of Blood Pool Agent 1 in Tumor A, but not in Tumor B **(Fig. 2E)**, indicating that LTX-315 disrupted the tumor vasculature at the site of injection only. Additionally, intravital imaging at the same time point revealed accumulation of Blood Pool Agent 2 in Tumor B, but not Tumor A **(Fig. 2E)**. Blood Pool Agent 1 and 2 co-localized in Tumor B, which confirmed that this control tumor remained vascularized as expected. By contrast, Blood Pool Agent 2 was undetectable in Tumor A, showing that its vasculature was profoundly impaired following LTX-315 treatment. These intravital imaging findings indicate that LTX-315 treatment can disrupt the tumor vasculature, which is otherwise essential to supply tumors with oxygen and nutrients.

### LTX-315's second phase of response

**Figure 3:**
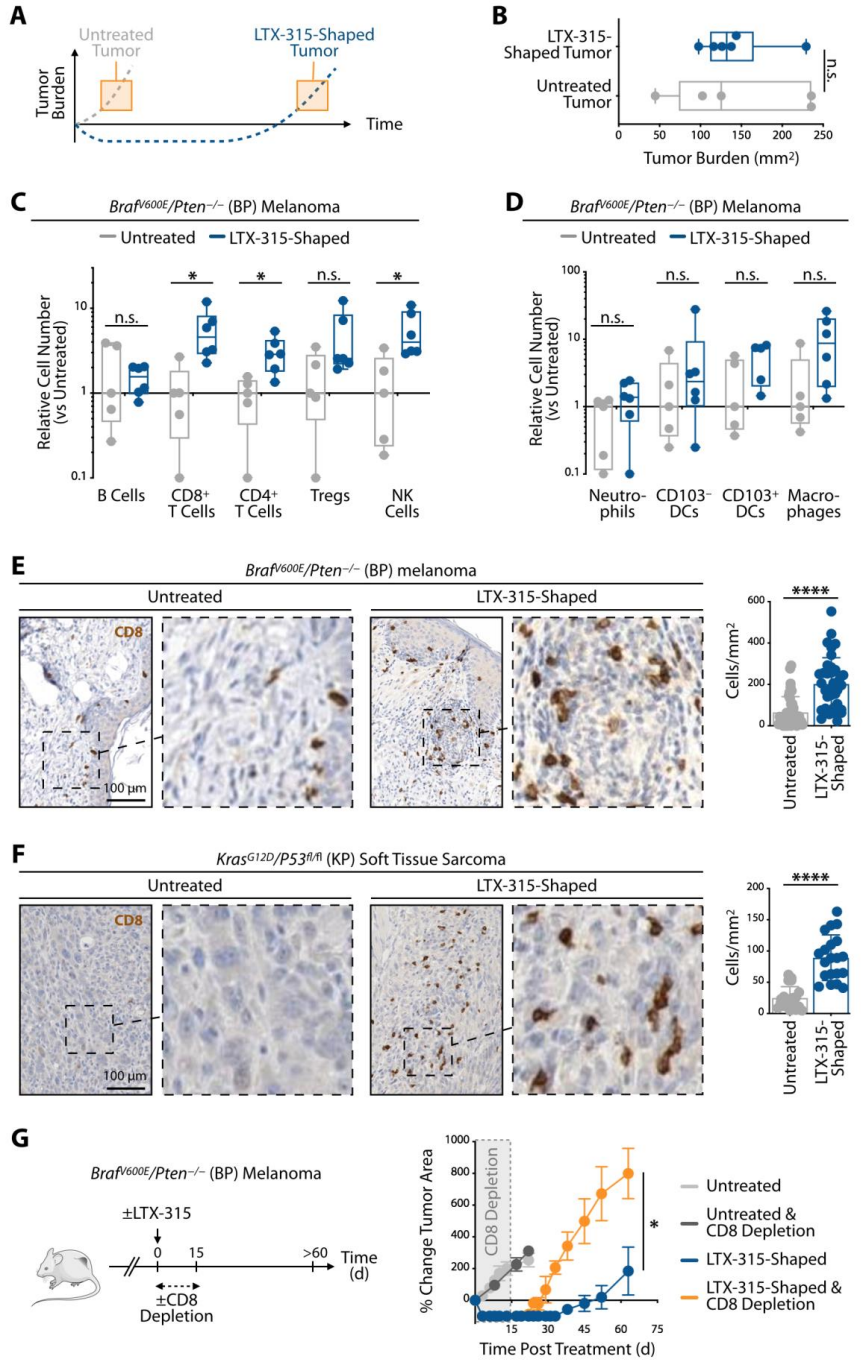
FIGURE 3: LTX-315's second phase of response is defined by long-term alteration of the tumor microenvironment including infiltration by antitumor CD8+ T cells. **(A)** Schematic of tumor growth curves of LTX-315 treated (blue) or untreated (grey) *Braf*^*V600E*^*/Pten*^*−/−*^ mice (BP model). Orange boxes highlight equal tumor burden in both groups and indicate the time points when tumor immune infiltrates were analyzed. **(B)** BP tumor area of LTX-315-treated and control mice with equal tumor burden as highlighted in (A); n = 5 to 6 mice/group. **(C)** *Ex vivo* flow cytometry-based evaluation of lymphocytes in tumors of LTX-315-treated BP mice or untreated mice at time points defined in (A). Data were normalized to control tumor-bearing mice. n = 5 to 6 mice/group. **(D)** Same analysis as shown in (C) but focusing on intratumoral myeloid cells. n = 5 to 6 mice/group. **(E)** Representative CD8 antibody staining and quantification on BP tumor tissue sections of untreated or LTX-315-treated mice. Samples were prepared at time points highlighted in (A) Scale bar, 100 μm. n = 3 mice/group, with 4-30 fields of view analyzed. **(F)** Same analysis as presented in (E) on *Kras*^*G12D*^*/p53*^*fl/fl*^ soft tissue sarcoma sections (KP model). Scale bar, 100 μm. n = 3-4 mice/group, with 10 fields of view analyzed. **(G)** Change in tumor area in either LTX-315-treated BP mice that received CD8 depleting antibodies for two weeks after drug injection or control mice (n = 3 to 6 mice/group). Results are expressed as mean ± SEM. *p < 0.05; ****p < 0.0001; n.s., not significant. Abbreviations are as follows: d = day.

Because LTX-315 treatment profoundly delayed melanoma re-growth in mice **(Fig. 1)**, we asked next whether tumors shaped by LTX-315 differ from those left untreated **(Fig. 3A)**. To this end, we compared untreated and LTX-315-shaped BP melanoma tumors, which were collected at different time points to be similar sized **(Fig. 3B)**. Tumors that regrew after LTX-315 treatment were macroscopically similar to tumors that were left untreated. Since we noted vascular disruption as an early antitumor effect of LTX-315 treatment, and considering that the vasculature also impacts immune cells infiltration, we also asked whether the effects on the tumor vasculature were still detectable in the second phase of response. Tissue sections obtained from either untreated or LTX-315-shaped BP tumors, and stained for CD31 to detect blood vessels, showed similar tumor vasculature, indicating that tumors that regrew after LTX-315 treatment were vascularized **(Fig. S2A)**. We extended these findings to the KP mouse model **(Fig. S2B)**.

Further analysis of lymphocyte content within these tumors showed unchanged accumulation of B cells. However, the number of CD8+ T cells, CD4+ T cells and natural killer (NK) cells significantly expanded in LTX-315-shaped tumors when compared to their untreated counterparts. The number of CD8+ T cells notably increased, with ~five times more in LTX-315-shaped tumors **(Fig. 3C)**. FOXP3+ CD4+ regulatory T cells may also be more numerous in LTX-315-shaped tumors although this increase did not reach significance. The number of myeloid cells, including neutrophils, dendritic cells and macrophages, remained largely unchanged **(Fig. 3D)**.

Clinical investigations have shown that tumor immune cell infiltration levels correlate with clinical outcomes in many cancers; remarkably, tumor infiltration by CD8 + T cells (“hot tumors”) is frequently associated with improved overall patient prognosis and response to treatment [16]. We thus sought to further evaluate CD8+ T cell accumulation by histology in tumors that received LTX-315 treatment. Analysis of BP melanomas showed significantly increased densities of CD8+ cells within LTX-315-shaped tumors, when compared to their untreated counterparts **(Fig. 3E)**. Similarly, the density of CD8+ cells also significantly increased within KP soft tissue sarcomas that received and responded to LTX-315 treatment **(Fig. 3F)**. These findings indicate LTX-315's ability to promote tumor infiltration by CD8+ T cells in different genetically induced cancer models.

LTX-315-induced accumulation of CD8+ T cells within tumors suggested the oncolytic peptide may promote antitumor immunity. To test this hypothesis in melanoma, mice carrying BP tumors and treated with LTX-315 received anti-CD8 depleting antibodies to reduce the number of CD8+ T cells during the 15 days following LTX-315 treatment **(Fig. 3G)**. Control groups included LTX-315-treated mice in which CD8+ T cells were not depleted as well as untreated mice that either received, or did not receive, the anti-CD8 depleting antibodies. CD8+ T cell depletion in untreated mice failed to alter BP tumor progression, which accords with the findings that these melanoma tumors are poorly infiltrated by CD8+ T cells and that tumor growth likely does not depend on CD8+ T cells. As expected, CD8+ T cell depletion in LTX-315-treated mice did not prevent initial BP tumor control in the days that followed LTX-315 administration (first phase of response); however, it triggered significantly faster tumor re-growth (second phase of response) **(Fig. 3G)**. These findings not only support the notion that initial tumor control is independent of lymphocytes (see also **Fig. 2)**, but also indicate that the CD8+ T cells accumulating in BP tumors in response to LTX-315 treatment have antitumor functions and are involved in suppressing cancer progression during the following weeks. Tumor growth in LTX-315-treated and CD8+ T cell depleted mice was perhaps more rapid than in untreated mice, further suggesting that the rate at which tumors regrow following LTX-315 treatment depends at least in part on CD8+ T cells.

### LTX-315's impact on CD8+ T cell accumulation in patients' tumors

**Figure 4:**
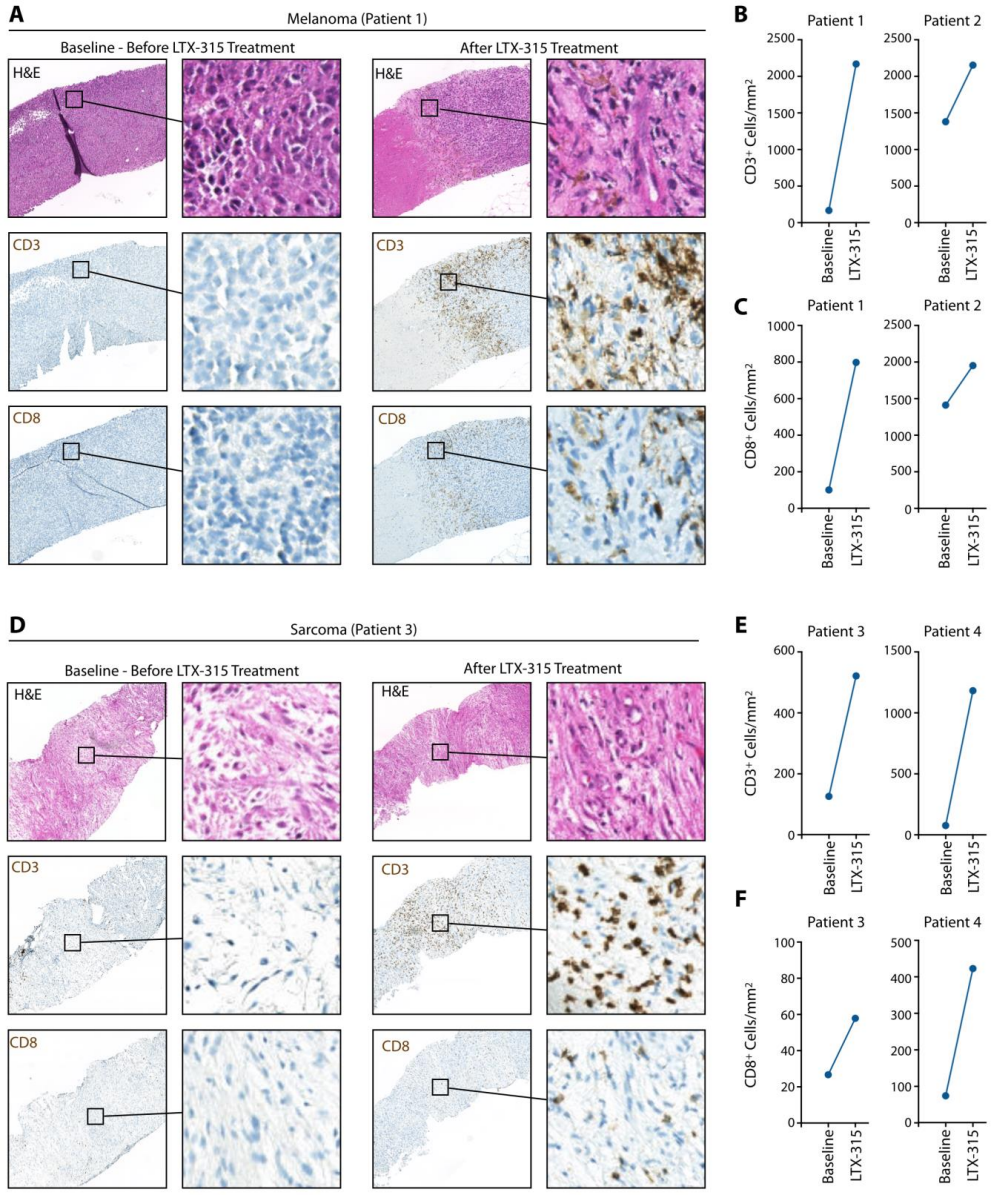
FIGURE 4: LTX-315 treatment causes CD8 T cell infiltration of melanoma and soft tissue sarcoma tumors in patients. **(A)** Representative hematoxylin and eosin (H&E) staining and immunohistochemistry for CD3^+^ and CD8^+^ cells (brown) on melanoma patient biopsy sections before and after LTX-315 treatment. **(B)** Quantification of CD3^+^ cells in melanoma biopsies, based on immunohistochemistry staining shown in (A). **(C)** Same analysis as shown in (B) for quantification of CD8^+^ cells. **(D)** Representative H&E staining and immunohistochemistry for CD3^+^ and CD8^+^ cells (brown) on patient biopsy sections of sarcomas before and after LTX-315 treatment. **(E)** Quantification of CD3^+^ cells in sarcoma biopsies, based on immunohistochemistry staining shown in (D). **(F)** Same analysis as shown in (E) for quantification of CD8^+^ cells.

Having identified LTX-315's ability to trigger CD8+ T cell accumulation in genetically induced mouse models of cancer, we extended our analyses to patients. We analyzed four of them, who were diagnosed with either melanoma or sarcoma, and received LTX-315 intratumorally (please see the Methods section for additional information on the patients and treatment regimen). Tumor biopsies were obtained before LTX-315 administration (Baseline) and four weeks (seven weeks for patient #4) after treatment and both specimens were evaluated by histology. These analyses identified increased tumor infiltration by CD3+ and CD8+ cells following LTX-315 treatment in both melanoma patients **(Fig. 4A-C)**. Similarly, we found increased tumor infiltration by CD3+ and CD8+ cells following LTX-315 in both sarcoma patients **(Fig. 4D-F)**. We conclude from these studies that LTX-315-triggered tumor infiltration by CD8+ T cells is not limited to experimental mouse models but can happen similarly in patients. The observation that CD8+ T cells accumulate in both human melanomas and sarcomas following LTX-315 treatment further suggests that the oncolytic peptide may be used to promote CD8+ T cell responses across cancer types.

## DISCUSSION

Our findings reveal that LTX-315 mediates multiple antitumor effects, including disruption of the tumor vasculature (phase 1) and conversion of weakly immunogenic tumors into ones that display antitumor T cell immunity (phase 2). Below we discuss our selection of tumor mouse models to study LTX-315, the two phases of response triggered by the oncolytic peptide in these models and this work's relevance for future experimental and clinical studies.

Animal models are useful for analyzing drug action mechanisms; however, their utility for studying human disease remains debatable. Here, to explore LTX-315's antitumor effects, we took care to select several tumor mouse models that feature keys aspects of the human disease. We studied B16F10-bearing mice, because they are one of the most commonly used experimental models to study melanoma and have already provided data for several clinical studies. Also, B16F10-bearing mice are helpful when evaluating new therapeutic strategies because they are poorly immunogenic, elicit a low frequency of tumor-specific T cells and are refractory to immune checkpoint therapy and other standard-of-care treatments [17]. In addition to B16F10, we used the *Braf*^*V600E*^*/Pten*^*−/−*^ (BP) model because melanoma tumors in this model carry mutations that are often found in the human disease and arise directly from somatic cells that are transformed in their normal tissue microenvironment [9]. Also, we studied BP tumors because we found that, like B16F10 tumors, they are poorly infiltrated by T cells and resist immune checkpoint therapy. Whereas this work focused mostly on melanoma, we also used *Kras*^*LSL-G12D/+*^*;p53*^*fl/fl*^ (KP) mice to extend some of our studies to another genetically induced cancer mouse model. Similarly to B16F10 and BP melanomas, KP soft tissue sarcomas are useful for studying LTX-315 antitumor effects, because these tumors are poorly infiltrated by T cells and resist current treatment options, including immune checkpoint therapy. Finally, as we aimed to understand LTX-315's effects on tumor microenvironments, we not only interrogated melanoma and sarcoma lesions in mice, but also extended our studies to patients with the same diseases and from whom tumor biopsies were collected both before and after LTX-315 intratumoral administration. The combination of these approaches allowed us to study the effects of LTX-315 on diverse tumor microenvironments and further define the relevance of using this agent in clinical trials.

The first phase of response triggered by LTX-315 began within minutes after drug delivery into tumors and was defined by disrupted tumor vasculature and decreased tumor burden. Considering that tumor cells receive essential oxygen and nutrients via diffusion from the vascular supply, these findings support the notion that LTX-315's direct effects on the tumor vasculature are critical to controlling tumor progression immediately after drug administration in the various experimental models that were tested. Similar tumor control triggered by LTX-315 treatment in mice deficient in T and B cells further indicates that this phenomenon is not elicited by, or dependent on, antitumor lymphocytes.

By contrast, the second phase of response triggered by LTX-315 remained over weeks and was defined by the accumulation of tumor-infiltrating CD8+ T cells that displayed antitumor functions. Importantly, we found that LTX-315-induced CD8+ T cell infiltration in both BP and KP mice, which are two tumor mouse models likely to carry a limited neoantigen load (particularly the KP model). Considering that tumors with fewer neoantigens are typically poorly infiltrated by CD8+ T cells and less likely to respond to immunotherapy [14], our findings suggest that LTX-315 treatment could have broad applicability including to treat tumors that fail to respond to current standard-of-care treatments. In addition to CD8+ T cells, we found that LTX-315 induced changes in other immune cell types in the tumor microenvironment, including CD4+ T cells and NK cells, which may also contribute important antitumor activities [18, 19].

In comparing the effects of LTX-315 treatment on different tumor mouse models, we observed increased effectiveness in the BP melanoma model compared to the KP sarcoma model. The particularly low mutational load of KP tumors [20] might make them less immunogenic when compared to BP tumors. Consequently, whereas the dependency on CD8^+^ T cell activity of the secondary anti-tumor response is a phenomenon that we observed in the BP melanoma model, it is possible that other mechanisms apply to other tumors, such as KP sarcomas in which the mutational load is low.

Both the oncolytic peptide LTX-315 and oncolytic viruses can be used to make “cold” tumors “hot”. Possible advantages of using LTX-315 are that this peptide does not induce an antibody response and can be injected repeatedly; it also has a short half-life and is thus degraded rapidly; and it can easily be handled and stored since it is not a genetically modified organism. Initial preclinical studies, which tested LTX-315 and oncolytic viruses in identical models, further suggest that at least in some experimental settings treatment with virus alone may only delay tumor growth whereas LTX-315 treatment could induce complete tumor regression. Furthermore, in a regimen combining either the virus or LTX-315 with anti-CTLA4, the LTX-315 was superior in controlling tumor growth [7, 21].

In the studies presented here, we aimed to examine some fundamental mechanisms triggered by LTX-315 therapy. From a translational perspective, further preclinical studies should help identify therapeutic approaches that control tumors longer. For example, it will be important to define in genetic mouse models whether re-applying LTX-315 to tumors that were initially controlled, but then regrew, could reinstate that control, or if acquired resistance to LTX-315 therapy emerges (and, if so, which phase(s) of the LTX-315 response is(are) altered).

It will also be important to determine which combination treatments may extend tumor control. Since LTX-315 triggers tumor infiltration by antitumor T cells, it is possible that drugs that further activate these cells, such as anti-PD-1 mAbs, may be beneficial at least in some settings when combined with LTX-315. Combining LTX-315 with myeloid cell-targeting agents [22–24] may also be useful. Myeloid cells can diversify in a spectrum of states, including some that promote cancer outgrowth and other that restrict it [22, 25]. The initial data presented here suggest that LTX-315 treatment does not alter the overall number of myeloid cells within tumors, but further knowing whether LTX-315 treatment influences the phenotype and function of (at least some) myeloid cells may help define new combination treatment options.

Different drug regimen schedules should also be considered since the sequence used to inject the different agents may affect treatment outcome. For example, LTX-315's capacity to disrupt the tumor vasculature indicates that other drugs, particularly those injected systemically, should be administered either before LTX-315, or sufficiently later, for efficient delivery within the tumor stroma. Interestingly, LTX-315 treatment, in offering the ability to convert non-T-cell-infiltrated tumors into ones that display antitumor T cell immunity, opens the possibility to prime and render unresponsive tumors sensitive to subsequent systemic immune treatments.

## MATERIALS AND METHODS

### Mice

*Kras*^*LSL-G12D/+*^*;p53*^*flox/flox*^ (KP) mice were used as a conditional mouse model of soft tissue sarcoma and bred in our laboratory in the C57BL/6 background. *Braf*^*V600E*^*;Pten*^*–/–*^ (BP) mice (B6.Cg-Braf^tm1Mmcm^ Pten^tm1Hwu^ Tg(Tyr-cre/ERT2)13Bos/BosJ) were used as a conditional mouse model of melanoma and bred in our laboratory in the FVB/N background. C57BL/6 wild type mice and *Rag2*^*–/–*^ mice were obtained from the Jackson Laboratory. All animal experiments were approved by the MGH Institutional Animal Care and Use Committee (IACUC) and were performed in accordance with MGH IACUC regulations.

### Cell lines

The melanoma cell line B16F10 was obtained from ATCC. The B16F10-GFP cell line was generated through stable transfection of B16F10 cells with GFP [26]. Both cell lines were cultured in Iscove's DMEM media supplemented with 10% fetal bovine serum (FBS) and 1% penicillin/streptomycin.

### Tumor models

To induce soft tissue sarcomas, KP mice were infected with an adenovirus expressing Cre recombinase (AdCre) by intramuscular injection to the leg, as described previously [10]. AdCre was purchased from the University of Iowa Gene Transfer Vector Core. Briefly, 50 μl of virus (2.5x10^7^ PFU/50 μl) diluted in MEM medium containing 2 M CaCl_2_ was injected per mouse. To induce melanomas, BP mice were injected with 4-hydroxytamoxifen (40 μg; 20 μg/μl stock solution) in 20 μl matrigel basement membrane matrix (Corning) in the interscapular area. B16F10 cells were implanted intradermally at 2x10^5^ cells in the flank of C57BL/6 or Rag2^*–/–*^ mice. Tumor area and leg sizes were measured with a digital caliper and calculated as length x width. Tumor volumes were defined as ½ (length x width^2^). Percent tumor changes or percent leg size changes were calculated as percent difference in mouse tumor area or tumor-bearing leg size from pre-treatment baseline. Mouse tumors were allowed to grow to a maximum of 2 cm in diameter, or until tumor ulceration occurred. These were considered endpoints for survival experiments in accordance with MGH IACUC regulations.

### *In Vivo* drug treatments

The peptide LTX-315 (1 mg in 50 μl Saline) from Lytix Biopharma was injected intratumorally when tumor sizes were approximately 25 mm^2^ (BP model), 20 mm^2^ (B16F10 model), or when leg sizes, defined by multiplying the two perpendicular dimensions of the leg at the largest cross section, were 15-50% larger than leg sizes of untreated mice (KP model). Typically, treatment of BP mice started around 28 days after 4-hydroxytamoxifen injection, treatment of KP mice started around 50 days after AdCre injection, whereas treatment of B16F10 treated mice started four days after tumor cell administration. For all tumor models, LTX-315 was injected for three consecutive days, with the first injection being defined as day 0 (see also Fig S1). In indicated experiments, immune checkpoint blockade antibodies specific for PD-1 (clone 29F.1A12, 250 μg/mouse, provided by Dr. G. J. Freeman) and CTLA-4 (clone 9D9, 100 μg/mouse, BioXcell) were applied intraperitoneally (i.p.) in 100 μl PBS. This regimen was used because they can be effective against tumors that are infiltrated by T cells [17, 27]. The antibodies were injected on day 0 and subsequently three times per week until the mice were euthanized.

### Intravital imaging

To analyze tumors' vascular integrity following LTX-315 injection, mice were anesthetized and dorsal skin-fold window chambers were installed as previously described [28, 29]. Mice were treated with analgesic (Buprenorphine 0.1 mg/kg/day) for three days following chamber implantation. 24 hours after window chamber implantation, 5x10^4^ B16F10-GFP cells (green) were injected in the fascia layer. In some experiments, two tumors (tumor A and tumor B) were implanted per mouse. For vascular labeling, a blood pool agent (Dextran-VT680, white) was delivered via a 30-gauge catheter inserted in the tail vein of the anesthetized mice (2% isoflurane in oxygen) one week after tumor implantation. Ten minutes later LTX-315 (1 mg in 50 μl saline) was injected intratumorally and mice were imaged for 45 min. In mice that carried two tumors, LTX-315 was injected into tumor A and both tumors (A and B) were imaged over time. 60 min after LTX-315 injection, a 30-gauge catheter inserted into the tail vein delivered a second blood pool agent (Dextran-TRITC, red) to visualize vascular integrity of both tumors following drug administration. During imaging, anesthetized mice were kept on a heating pad set to 37°C, vital signs were monitored and mice were imaged using an Olympus FluoView FV1000MPE confocal imaging system (Olympus America). Z-stack images were processed using the FIJI MacOS bundle of ImageJ (version 1.8.0_101), as previously described [30].

### Flow cytometry

Single-cell suspensions were prepared from tumor tissue of LTX-315-treated or untreated mice for flow cytometry analysis. Tumors were harvested, cut into small pieces using scissors and digested with collagenase type I (0.2 mg/ml, Worthington Biochemical Corporation) in RPMI 1640 medium for 30 min at 37°C while shaking. Digested tumor tissue was gently meshed through 40 μM cell strainer using a plunger. Red blood cells were removed by Ack lysis buffer (Lonza) according to the manufacturer's instructions. The resulting single-cell suspensions were washed with PBS and stained with the zombie aqua fixable viability kit (Biolegend) for 20 min at room temperature to exclude dead cells. Next, cells were washed with staining buffer (PBS containing 0.5% BSA and 2 mM EDTA) and incubated with Fc Block (clone 93, BioLegend) for 15 min at 4°C followed by staining with fluorescent conjugated antibodies for 45 min at 4°C. Doublet cells were excluded based on their forward/side scatter properties by flow cytometry analysis (LSRII, BD). The following cell populations were identified based on cell marker expression: B cells (CD45^+^ CD19^+^), CD8^+^ T cells (CD45^+^ CD3^+^ CD8^+^), CD4^+^ T cells (CD45^+^ CD3^+^ CD4^+^), Tregs (CD45^+^ CD3^+^ CD4^+^ FOXP3^+^), NK cells (CD45^+^ NKp46^+^), neutrophils (CD45^+^ CD11b^+^ Ly-6G^+^), CD103^–^ DCs (CD45^+^ CD11b^+^ Ly-6G^–^ CD11c^+^ CD103^–^), CD103^+^ DCs (CD45^+^ CD11b^+^ Ly-6G^–^ CD11c^+^ CD103^+^), macrophages (CD45^+^ CD11b^+^ F4/80^+^ CD11c^+^). The following antibodies were purchased from BD: CD4 (clone RM4-5), CD19 (clone 1D3), CD11b (clone M1/70), CD49b (clone DX5), Ly-6G (clone 1A8), CD4 (clone RM4-5); from eBioscience: Foxp3 (clone FJK-16s) and from Biolegend: CD11c (clone N418), F4/80 (clone BM8), CD45 (clone 30-F11), CD3e (clone 145-2C11), CD8 (clone 53.-6.7), NKp46 (clone 29A1.4) and CD103 (clone 2E7). Intracellular staining for FOXP3 was performed using the Cytofix/Cytoperm Fixation/Permeabilization Kit (BD) according to the manufacturer's procedures. Flow cytometry data were analyzed in FlowJo (Tree Star, Inc.).

### Immunohistochemistry on mouse samples

For histological analysis, tumor tissues were fixed in formaldehyde and embedded in paraffin following standard procedures and consecutive sections were prepared. Immunohistochemistry on mouse tissue sections was performed as previously described [31]. Briefly, mouse tumor sections were prepared using a Leica RM2255 rotary microtome (Leica Biosystems), dried at 60°C for 1 h, dewaxed and rehydrated before heat-induced epitope-retrieval (HIER) prior to immunostaining. The sections were incubated in 10 mM sodium-citrate (pH 6.0) buffered solution containing 0.05% Tween at 120°C for 2 min using a pressure cooker. To obtain consistent and reliable staining results the LabVision Autostainer 360 (Thermo Fisher Scientific) was used. To destroy endogenous peroxidase activity, the sections were pretreated using BLOXALL endogenous enzyme blocking solution (Vector Laboratories) for 10 min. After blocking with normal goat serum, the sections were incubated with rat anti-mouse CD8a antibodies (clone 4SM15, eBiosciences) for 1 h, followed by several washes and secondary ImmPRESS polymer detection system (Vector Laboratories) according to the manufacturer's protocol. DAB Quanto (Thermo Fisher Scientific) was used as substrate and hematoxylin as counterstain. The tumor vasculature was detected with Rabbit polyclonal Ab to CD31 (ab28364, Abcam). Image documentation was performed using the NanoZoomer 2.0-RS slide scanner system (Hamamatsu) and CD8 positivity was identified using Fiji software. The counting results are expressed as cells/mm^2^.

### *In Vivo* CD8^+^ cell depletion

To deplete CD8^+^ T cells, BP mice received anti-CD8a monoclonal antibodies i.p. (clone 53-6.72, 200 μg/mouse, BioXcell). The antibody was diluted in PBS. Injections were started two days before the first LTX-315 drug injection and were continued every 2-3 days for 2 weeks.

### Human tumor samples

The Regional Committee for Medical and Health Research Ethics (REK-Sør-Øst) approved the use of human material for this study (Project-ID: 2016/1650/REK Sør-Øst). Tumor biopsies were obtained from melanoma and sarcoma patients before LTX-315 treatment and four weeks post treatment, except for patient #4 who had biopsy seven weeks post treatment. Patient #1, a 71 years old female, was diagnosed with nodular type melanoma in March 2011. Prior treatments included wide excision of tumor (March 2011), pembrolizumab (December 2012, partial response), ipilimumab (February 2016, partial response), and radiotherapy of parietal area (December 2016) and orbit (May 2017). The patient started a 3-week LTX-315 treatment in August 2017, which consisted of six injections (3 mg per injection, or total dose of 18 mg) in one lesion. Patient #2, a 71 years old male, was diagnosed with uveal melanoma in June 2014. Prior treatments included radiotherapy (June 2014); vitrectomy (September 2014); deticene (October 2015, progressive disease); nivolumab (November 2015, partial response). The patient started a 3-week LTX-315 treatment in February 2018, which consisted of 24 injections (5 mg per injection, or total dose 120 mg) in one lesion. Patient #3, a 63 years old female, was diagnosed with soft tissue sarcoma in her thigh (malignant peripheral nerve sheath tumor) in January 2007. Prior treatments included ifosfamide and doxorubicin (February 2007); radiotherapy to right thigh (April 2007); pelvic excision of lesion (June 2007); hemipelvectomy (September 2009); ifosfamide and doxorubicin (March 2013, stable disease); resection of thoracic wall, diaphragm, pericardium (April 2013); gemcitabine and docetaxol (November 2013, progressive disease); sorafenib (January 2014, progressive disease); carboplatin and etoposide (February 2015, stable disease); carboplatin and etoposide (September 2016, stable disease). The patient started a 3-week LTX-315 treatment in May 2017, which consisted of 18 injections (3 mg per injection, or total dose of 54 mg) in one lesion. Patient #4, a 30 years old female, was diagnosed with desmoid sarcoma in thorax in January 2013. Prior treatment included interferon (May 2014, stable disease). The patient started a 6-week induction LTX-315 treatment in May 2017, followed by a 9-week maintenance period. The treatment consisted of 13 injections (4 mg first injection, 5 mg in the following injections or 59 mg) in one lesion.

### Immunohistochemistry on human samples

Formalin-fixed, paraffin-embedded tissue slides were deparaffinized and rehydrated in graded alcohols. Staining was performed on two consecutive slides using a qualified Benchmark XT associated with the following workflow and selected reagents: For antigen retrieval, slides were boiled in a sodium citrate buffer (pH 6.0) for 10 min. Sections were immunostained with anti-human CD3 and anti-human CD8, respectively (clones from HalioDx). To visualize immunopositive staining, sections were labeled with secondary antibody followed by DAB-peroxidase substrate solution. Sections were then counterstained with Haematoxylin and mounted. A routine standard staining was performed with hematoxylin and eosin. Each slide was scanned with the Nanozoomer XR / x20. Quantification of CD3 and CD8 positive cells was done using HalioDx Digital Pathology Platform. Raw data included CD3 and CD8 density into the core tumor (in cells/mm^2^).

### Statistical analysis

All statistical analyses were performed using Graphpad Prism Version 7. Results were expressed as mean±SEM. Unpaired t test was done to compare two groups. One-way or Two-way ANOVA with subsequent post-hoc analysis was used to compare three or more groups. For Kaplan-Meier survival analysis, p values were computed using the Log Rank Mantel-Cox test. p values > 0.05 were considered not significant (n.s.); p values < 0.05 were considered significant. * p value < 0.05, ** p value < 0.01, *** p value < 0.001, **** p value < 0.0001.

## SUPPLEMENTAL MATERIAL

Click here for supplemental data file.

All supplemental data for this article are also available online at http://www.cell-stress.com/researcharticles/2019a-liao-cell-stress/.

## Abbreviations:

BP: – Braf^V600E^/Pten^−/−^,
GFP: – green fluorescence protein,
KP: – Kras^LSL-G12D/+^;p53^fl/fl^,
NK: – natural killer.

## References

[1] Haug BE, Camilio KA, Eliassen LT, Stensen W, Svendsen JS, Berg K, Mortensen B, Serin G, Mirjolet JF, Bichat F, Rekdal Ø (2016). Discovery of a 9-mer Cationic Peptide (LTX-315) as a Potential First in Class Oncolytic Peptide.. J Med Chem.

[2] Zhou H, Forveille S, Sauvat A, Sica V, Izzo V, Durand S, Müller K, Liu P, Zitvogel L, Rekdal Ø, Kepp O, Kroemer G (2015). The oncolytic peptide LTX-315 kills cancer cells through Bax/Bak-regulated mitochondrial membrane permeabilization.. Oncotarget.

[3] Eike LM, Yang N, Rekdal Ø, Sveinbjørnsson B (2015). The oncolytic peptide LTX-315 induces cell death and DAMP release by mitochondria distortion in human melanoma cells.. Oncotarget.

[4] Galluzzi L, Buqué A, Kepp O, Zitvogel L, Kroemer G (2017). Immunogenic cell death in cancer and infectious disease.. Nat Rev Immunol.

[5] Camilio KA, Rekdal O, Sveinbjörnsson B (2014). LTX-315 (Oncopore™): A short synthetic anticancer peptide and novel immunotherapeutic agent.. Oncoimmunology.

[6] Camilio KA, Berge G, Ravuri CS, Rekdal O, Sveinbjørnsson B (2014). Complete regression and systemic protective immune responses obtained in B16 melanomas after treatment with LTX-315.. Cancer Immunol Immunother.

[7] Yamazaki T, Pitt JM, Vétizou M, Marabelle A, Flores C, Rekdal Ø, Kroemer G, Zitvogel L (2016). The oncolytic peptide LTX-315 overcomes resistance of cancers to immunotherapy with CTLA4 checkpoint blockade.. Cell Death Differ.

[8] Nestvold J, Wang MY, Camilio KA, Zinöcker S, Tjelle TE, Lindberg A, Haug BE, Kvalheim G, Sveinbjørnsson B, Rekdal Ø (2017). Oncolytic peptide LTX-315 induces an immune-mediated abscopal effect in a rat sarcoma model.. Oncoimmunology.

[9] Dankort D, Curley DP, Cartlidge RA, Nelson B, Karnezis AN, Damsky WE, You MJ, Depinho RA, Mcmahon M, Bosenberg M (2009). Braf(V600E) cooperates with Pten loss to induce metastatic melanoma.. Nat Genet.

[10] Kirsch DG, Dinulescu DM, Miller JB, Grimm J, Santiago PM, Young NP, Nielsen GP, Quade BJ, Chaber CJ, Schultz CP, Takeuchi O, Bronson RT, Crowley D, Korsmeyer SJ, Yoon SS, Hornicek FJ, Weissleder R, Jacks T (2007). A spatially and temporally restricted mouse model of soft tissue sarcoma.. Nat Med.

[11] Leimgruber A, Berger C, Cortez-Retamozo V, Etzrodt M, Newton AP, Waterman P, Figueiredo JL, Kohler RH, Elpek N, Mempel TR, Swirski FK, Nahrendorf M, Weissleder R, Pittet MJ (2009). Behavior of endogenous tumor-associated macrophages assessed in vivo using a functionalized nanoparticle.. Neoplasia.

[12] Gajewski TF, Schreiber H, Fu YX (2013). Innate and adaptive immune cells in the tumor microenvironment.. Nat Immunol.

[13] Joyce JA, Fearon DT (2015). T cell exclusion, immune privilege, and the tumor microenvironment.. Science.

[14] Schumacher TN, Schreiber RD (2015). Neoantigens in cancer immunotherapy.. Science.

[15] Sveinbjørnsson B, Camilio KA, Haug BE, Rekdal Ø (2017). LTX-315: a first-in-class oncolytic peptide that reprograms the tumor microenvironment.. Future Med Chem.

[16] Fridman WH, Pagès F, Sautès-Fridman C, Galon J (2012). The immune contexture in human tumours: impact on clinical outcome.. Nat Rev Cancer.

[17] Garris CS, Arlauckas SP, Kohler RH, Trefny MP, Garren S, Piot C, Engblom C, Pfirschke C, Siwicki M, Gungabeesoon J, Freeman GJ, Warren SE, Ong S, Browning E, Twitty CG, Pierce RH, Le MH, Algazi AP, Daud AI, Pai SI, Zippelius A, Weissleder R, Pittet MJ (2018). Successful Anti-PD-1 Cancer Immunotherapy Requires T Cell-Dendritic Cell Crosstalk Involving the Cytokines IFN-γ and IL-12.. Immunity.

[18] Borst J, Ahrends T, Bąbała N, Melief CJM, Kastenmüller W (2018). CD4^+^ T cell help in cancer immunology and immunotherapy.. Nat Rev Immunol.

[19] Morvan MG, Lanier LL (2016). NK cells and cancer: you can teach innate cells new tricks.. Nat Rev Cancer.

[20] Lee CL, Mowery YM, Daniel AR, Zhang D, Sibley AB, Delaney JR, Wisdom AJ, Qin X, Wang X, Caraballo I, Gresham J, Luo L, Van Mater D, Owzar K, Kirsch DG (2019). Mutational landscape in genetically engineered, carcinogen-induced, and radiation-induced mouse sarcoma.. JCI Insight.

[21] Fend L, Yamazaki T, Remy C, Fahrner C, Gantzer M, Nourtier V, Préville X, Quéméneur E, Kepp O, Adam J, Marabelle A, Pitt JM, Kroemer G, Zitvogel L (2017). Immune Checkpoint Blockade, Immunogenic Chemotherapy or IFN-α Blockade Boost the Local and Abscopal Effects of Oncolytic Virotherapy.. Cancer Res.

[22] Engblom C, Pfirschke C, Pittet MJ (2016). The role of myeloid cells in cancer therapies.. Nat Rev Cancer.

[23] Coffelt SB, Wellenstein MD, De Visser KE (2016). Neutrophils in cancer: neutral no more.. Nat Rev Cancer.

[24] Denardo DG, Ruffell B (2019). Macrophages as regulators of tumour immunity and immunotherapy.. Nat Rev Immunol.

[25] Zilionis R, Engblom C, Pfirschke C, Savova V, Zemmour D, Saatcioglu HD, Krishnan I, Maroni G, Meyerovitz CV, Kerwin CM, Choi S, Richards WG, De Rienzo A, Tenen DG, Bueno R, Levantini E, Pittet MJ, Klein AM (2019). Single-Cell Transcriptomics of Human and Mouse Lung Cancers Reveals Conserved Myeloid Populations across Individuals and Species.. Immunity.

[26] Pucci F, Garris C, Lai CP, Newton A, Pfirschke C, Engblom C, Alvarez D, Sprachman M, Evavold C, Magnuson A, Von Andrian UH, Glatz K, Breakefield XO, Mempel TR, Weissleder R, Pittet MJ (2016). SCS macrophages suppress melanoma by restricting tumor-derived vesicle-B cell interactions.. Science.

[27] Pfirschke C, Engblom C, Rickelt S, Cortez-Retamozo V, Garris C, Pucci F, Yamazaki T, Poirier-Colame V, Newton A, Redouane Y, Lin YJ, Wojtkiewicz G, Iwamoto Y, Mino-Kenudson M, Huynh TG, Hynes RO, Freeman GJ, Kroemer G, Zitvogel L, Weissleder R, Pittet MJ (2016). Immunogenic Chemotherapy Sensitizes Tumors to Checkpoint Blockade Therapy.. Immunity.

[28] Arlauckas SP, Garris CS, Kohler RH, Kitaoka M, Cuccarese MF, Yang KS, Miller MA, Carlson JC, Freeman GJ, Anthony RM, Weissleder R, Pittet MJ (2017). In vivo imaging reveals a tumor-associated macrophage-mediated resistance pathway in anti-PD-1 therapy.. Sci Transl Med.

[29] Pittet MJ, Garris CS, Arlauckas SP, Weissleder R (2018). Recording the wild lives of immune cells.. Sci Immunol.

[30] Arlauckas SP, Garren SB, Garris CS, Kohler RH, Oh J, Pittet MJ, Weissleder R (2018). Arg1 expression defines immunosuppressive subsets of tumor-associated macrophages.. Theranostics.

[31] Engblom C, Pfirschke C, Zilionis R (2017). Osteoblasts remotely supply lung tumors with cancer-promoting SiglecF^high^ neutrophils.. Science.

