# Supplementary material for: LTX-315 sequentially promotes lymphocyte-independent and lymphocyte-dependent antitumor effects
Source: Cell Stress. 2019 Oct 14;3(11):348–60. doi: 10.15698/cst2019.11.204 (PMC6859426; doi:10.15698/cst2019.11.204)
Supplement: Supplementary file 1 [file ces-03-348-s01.pdf]

**Supplemental Information**

# LTX-315 sequentially promotes lymphocyte-independent and lymphocyte-dependent antitumor effects

Hsin-Wei Liao[1*], Christopher Garris[1*], Christina Pfirschke[1], Steffen Rickelt[2], Sean Arlauckas[1], Marie Siwicki[1], Rainer Kohler[1], Ralph Weissleder[1], Vibeke Sundvold-Gjerstad[3], Baldur Sveinbjørnsson[3,4], Øystein Rekdal[3,4], Mikael J. Pittet[1•]

[1]Center for Systems Biology, Massachusetts General Hospital Research Institute, Harvard Medical School, Boston, MA, USA; [2]David H. Koch Institute for Integrative Cancer Research, Massachusetts Institute of Technology, Cambridge, MA, USA; [3]Lytix Biopharma, Oslo, Norway; [4]Department of Medical Biology, University of Tromsø, Tromsø, Norway.

*These authors contributed equally to this work; •Corresponding author

**Content**

Two Supplemental Figures: Fig S1, Fig S2

Fig S1

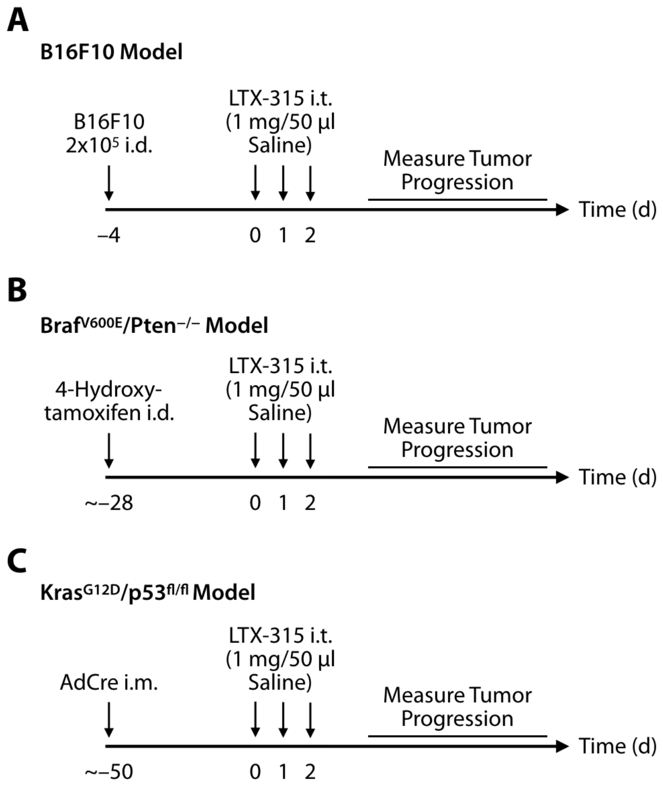
A
B16F10 Model
B16F10
2x10⁵ i.d.
LTX-315 i.t.
(1 mg/50 µl
Saline)
Measure Tumor
Progression
Time (d)
–4
0 1 2
B
Braf$^{V600E}$/Pten$^{-/-}$ Model
4-Hydroxy-
tamoxifen i.d.
LTX-315 i.t.
(1 mg/50 µl
Saline)
Measure Tumor
Progression
Time (d)
~–28
0 1 2
C
Kras$^{G12D}$/p53$^{fl/fl}$ Model
AdCre i.m.
LTX-315 i.t.
(1 mg/50 µl
Saline)
Measure Tumor
Progression
Time (d)
~–50
0 1 2

Fig S2

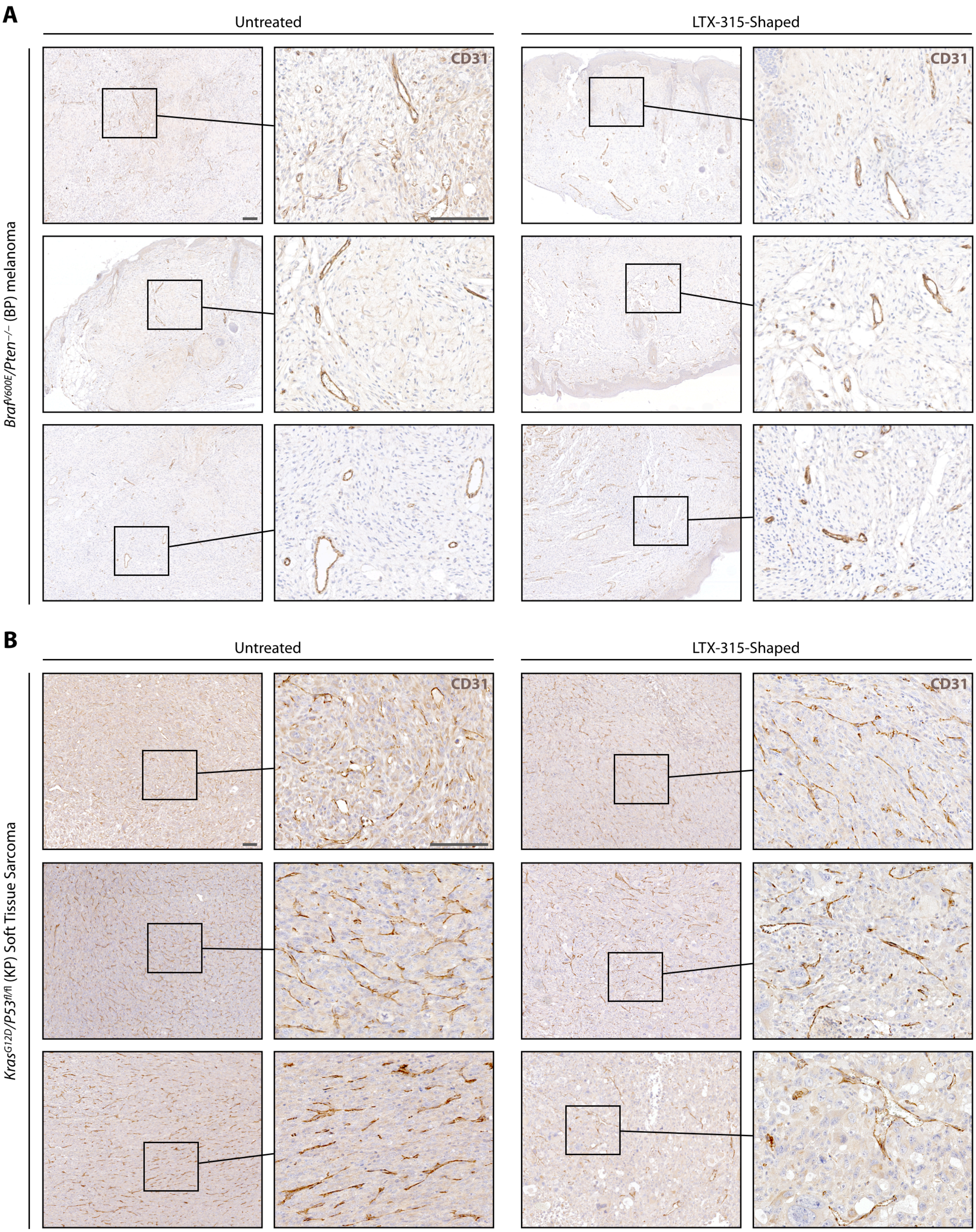
A
Untreated
LTX-315-Shaped
CD31
Braf$^{V600E}$/Pten$^{-/-}$ (BP) melanoma
B
Untreated
LTX-315-Shaped
CD31
Kras$^{G12D}$/P53$^{fl/fl}$ (KP) Soft Tissue Sarcoma

**Figure S1. LTX-315 treatment strategy in syngeneic and conditional genetic mouse models.**

(A) Schematic of B16F10 melanoma experiments: C57BL/6 wild type mice or *Rag2*$^{-/-}$ mice bearing B16F10 melanoma tumor grafts were treated intratumorally (i.t.) with LTX-315 for three consecutive days. Tumor-bearing control mice were left untreated.

(B) Schematic of BP melanoma experiments: *Braf*$^{V600E}$/*Pten*$^{-/-}$ (BP) mice subjected to tamoxifen to produce tumors were treated intratumorally (i.t.) with LTX-315 for three consecutive days or left untreated.

(C) Schematic of KP soft tissue sarcoma experiments: *Kras*$^{G12D}$/*p53*$^{fl/fl}$ (KP) mice subjected to intramuscular leg injection with Adenovirus expressing Cre recombinase (AdCre) to produce tumors were treated intratumorally (i.t.) with LTX-315 for three consecutive days. Tumor-bearing control mice were left untreated.

**Figure S2. CD31 staining in untreated and LTX-315-shaped BP and KP tumors.**

(A) CD31 staining of tumor sections obtained from *Braf*$^{V600E}$/*Pten*$^{-/-}$ (BP) mice that were either left untreated ("Untreated") or that regrew after LTX-315 treatment ("LTX-315-Shaped"). Both mouse cohorts had similar tumor burden (see also Figure 3A). Scale bar: 100 µm.

(B) CD31 staining of tumor sections obtained from *Kras*$^{G12D}$/*p53*$^{fl/fl}$ (KP) mice that were either left untreated ("Untreated") or that regrew after LTX-315 treatment ("LTX-315-Shaped"). Both mouse cohorts had similar tumor burden (see also Figure 3A). Scale bar: 100 µm.
